# Molecular insights into the role of Estrogen Receptor Beta in Ecdysterone Mediated Anabolic Activity

**DOI:** 10.1371/journal.pone.0320865

**Published:** 2025-06-02

**Authors:** Syeda Sumayya Tariq, Madiha Sardar, Muhammad Shafiq, Hendrick Heinz, Mohammad Nur-e-Alam, Aftab Ahmed, Zaheer Ul-Haq

**Affiliations:** 1 Dr. Panjwani Center for Molecular Medicine and Drug Research, International Center for Chemical and Biological Sciences, University of Karachi, Karachi, Pakistan; 2 H.E.J. Research Institute of Chemistry, International Center for Chemical and Biological Sciences, University of Karachi, Karachi, Pakistan; 3 Department of Chemical and Biological Engineering, University of Colorado at Boulder, Boulder, Colorado, United States of America; 4 Department of Pharmacognosy, College of Pharmacy, King Saud University, Riyadh, Kingdom of Saudi Arabia; 5 Department of Biomedical and Pharmaceutical Sciences, Chapman University School of Pharmacy, Irvine, California, United States of America; Kwara State University, NIGERIA

## Abstract

Ecdysterone, often dubbed a “natural steroid,” has garnered significant attention among athletes for its reputed growth-promoting and anabolic properties. Unlike synthetic anabolic steroids, which are classified as controlled substances, ecdysteroids remain largely unregulated in many countries and are widely marketed as dietary supplements. Notably, ecdysterone has been included in the World Anti-Doping Agency (WADA) monitoring program, highlighting its potential impact on athletic performance and raising questions about its regulation. Emerging evidence indicates that, unlike traditional anabolic steroids that act primarily via the Androgen Receptor (AR), ecdysterone’s anabolic effects may be mediated through Estrogen Receptors (ERs), particularly Estrogen Receptor beta (ERβ). Despite these insights, the precise molecular mechanisms underlying ecdysterone’s biological activity remain poorly characterized, particularly from an in-silico perspective. This paper aims to address these gaps by exploring ecdysterone’s mechanism of action through computational and molecular modeling approaches. This study employs an advanced computational framework to unravel the binding dynamics and interaction mechanisms of ecdysterone with Androgen Receptor (AR), Estrogen Receptor alpha (ERα), and Estrogen Receptor beta (ERβ). Using chemical descriptor analysis, inter-molecular interaction mapping, and all-atom molecular dynamics simulations spanning 250 ns for each system, the study reveals that ecdysterone preferentially binds to ERβ, forming stable and compact complexes characterized by minimal per-residue fluctuations as evident in the average RMSD, RMSF, and Rg values observed for ERβ - Ecdysterone as 1.98 ± 0.31 Å, 1.07 ± 0.52 Å, and 18.44 ± 0.08 Å respectively which are significantly comparable with the ERβ - native complex, while high hydrogen bond occupancy was also observed for ERβ - Ecdysterone complex. Although binding free energy calculations suggest stronger interactions with ERα, the associated high fluctuations diminish its binding efficacy. In contrast, interactions with ERβ remain consistent and robust. Machine learning-based principal component analysis highlights coordinated motion patterns, while free energy profiles demonstrate stable energy basins with minimal variation. These findings underscore the pivotal role of ERβ in mediating ecdysterone’s anabolic effects, distinguishing it from traditional androgenic steroids, and provide critical insights into its unique mechanism of action. This work lays the foundation for further exploration of ecdysterone as a potential anabolic agent.

## 1. Introduction

Ecdysteroids are a group of polar, polyhydroxylated steroid hormones essential for the growth, development, and reproduction of arthropods and are also found in high concentrations in plants termed as phytoecdysteroid. Extensive research has explored their potential growth-promoting and other beneficial effects in animals and humans. Unlike anabolic steroids, Ecdysteroids are believed to promote muscle growth and enhance physical performance without the severe side effects commonly associated with synthetic steroids, making them particularly appealing to athletes [1]. Ecdysterone, the most potent phytoecdysteroid, is increasingly popular among athletes for its claimed anabolic properties. It is widely marketed as a ‘natural’ dietary supplement, promising to enhance strength, muscle mass, reduce fatigue, and aid recovery during resistance training [2]. Unlike anabolic steroids, which are controlled substances, ecdysteroids are not classified as such in many countries and are legally available as dietary supplements. Ecdysteroids are not currently on the World Anti-Doping Agency (WADA) list of banned substances, but in 2020, Ecdysterone was added to the WADA monitoring program and is currently under investigation [3]

While research, particularly in animal studies and some human trials, supports Ecdysterone’s effects on muscle growth and performance, the precise mechanisms, especially in humans, are still under investigation. Ecdysterone, like other ecdysteroids, exerts its anabolic effects primarily through interaction with nuclear receptors, though its mechanism differs from that of traditional anabolic steroids. The significant structural differences between ecdysteroids and anabolic-androgenic steroids may account for their distinct anabolic mechanisms [4,5]. Recent in-vitro and in-vivo experimental studies conducted by Maria Kristina and colleagues suggest that Ecdysterone exerts its anabolic effects by binding to the estrogen (beta) receptor [6,7,8]. This study aims to bridge the gap by conducting a detailed exploration, specifically focusing on state-of-the-art in-silico methods for analyzing the experimental findings.

**Fig 1:**
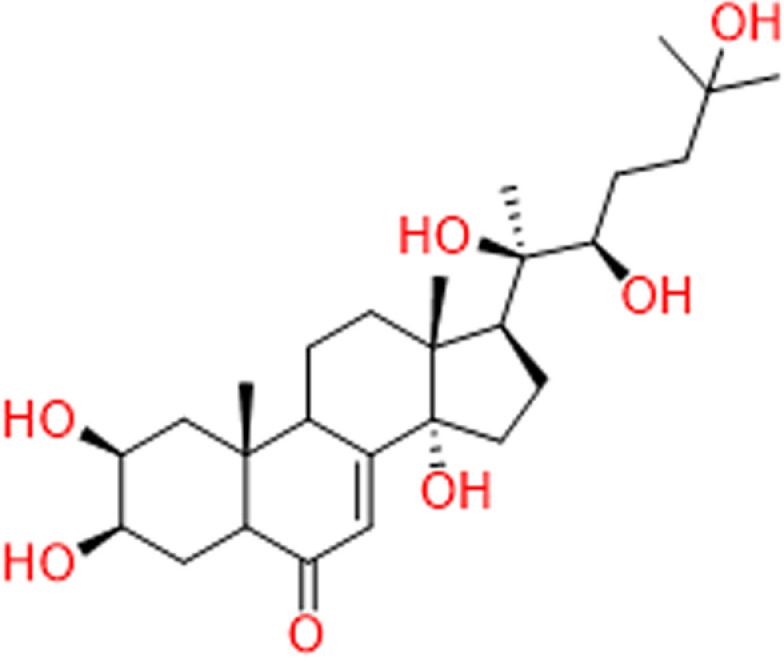
Structure of Ecdysterone.

In this context, this study is aimed at the in-silico exploration of the binding and interactions of Ecdysterone (Fig 1) with Androgen Receptor (AR), Estrogen Receptor alpha (ERα) and Estrogen Receptor beta (ERβ) in order to explore their atomic-level effects. For this purpose, a multifaceted computational framework was employed, involving in-silico techniques such as molecular docking to explore binding sites, and DFT techniques to calculate electrostatic potential and reactivity descriptors. Additionally, an all-atom molecular dynamics simulation was used to assess stability by examining deviation patterns, local flexibility shifts, time-dependent gyration profiles, and calculating free binding energies. To gain a deeper understanding of the dynamics and interactions within protein-ligand complexes, principal component analysis (PCA) was conducted in order to highlight essential features of the complex datasets produced by molecular dynamics (MD) simulations. Subsequently, Free Energy Landscapes (FEL) were also generated to reveal thermodynamic variations in the protein structure. This comprehensive analysis aims to deepen our understanding of the molecular interactions between Ecdysterone and AR, ERα, and ERβ, providing a foundation for clarifying its suitability for use in sports.

## 2. Methodology

### 2.1. Ligand preparation

Ecdysterone, the most potent phytoecdysteroid, was chosen for the current study leveraging on the experimental validation of its anabolic properties [2]. The chemical structures of Ecdysteroid and the native ligands of AR, ERα, and ERβ as identified in S1 Fig, were generated using ChemDraw [9] software. The MMFF94 [10] force field included in the Molecular Operating Environment (MOE) [11] package was used to supply hydrogen, minimize energy, and apply partial charges to these compounds. These preliminary actions were performed in order to derive spatial coordinates for each molecule that correspond to the most energetically favorable configuration. Subsequent docking studies used the optimized molecules as input files.

### 2.2. Protein preparation

This study is focused on three key proteins: Androgen Receptor (AR), Estrogen Receptor alpha (ERα), and Estrogen Receptor beta (ERβ). The structures of these proteins, retrieved from the Protein Data Bank with the PDB IDs 2YHD [12], 5WGD [13] and 4J26 [14] respectively, to serve as the preliminary point for further analysis. These PDB structures were selected based on several criteria, including the crystal structure’s resolution, the presence of the native ligand within the complex, and the year of their release. In the course of the protein preparation phase, several modifications were made to the structures, such as adding missing atoms and residues, adjustment of bond orders and formal charges. The protonate 3D [15] algorithm was used to incorporate hydrogen atoms. For protonation, the electrostatics function employed the generalized Born volume integral (GB/VI) method [16], with a solvent dielectric value of 80. Van der Waals and electrostatic cutoffs were set at 10 Å and 15 Å, respectively. The MOE software suite’s AMBER99 force field was used to apply partial charges.

### 2.3. Molecular Docking

The 3D structures of the Androgen Receptor (AR), Estrogen Receptor alpha (ERα), and Estrogen Receptor beta (ERβ) were imported into the MOE-Dock interface for docking simulations. Before initiating the docking studies, the software underwent benchmarking by comparing the root mean square deviation (RMSD) values between the crystal structure coordinates of the proteins’ native ligands and their corresponding simulated poses. This step, aimed at reproducing known binding geometries, validated the reliability of the docking protocol for subsequent analyses. The docking simulations utilized the Triangle Matcher algorithm for ligand placement, combined with the London dG scoring function and an Induced Fit approach to account for receptor flexibility. For each protein, ten conformations were generated for every metabolite of the studied organophosphate flame retardants (OPFRs), while retaining default parameter settings. The conformation with the lowest docking score, indicating the strongest binding affinity was then selected for detailed analysis of its binding orientation and interaction profile with AR, ERα, and ERβ.

### 2.4. Density Functional Theory (DFT)

To study the electronic properties of Ecdysterone, all-electron hybrid DFT calculations were conducted utilizing the CP2K software package [17] with the 6-31G(d) basis set and the B3LYP exchange-correlation functional, while maintaining an adequate energy cutoff at 450 Ry for the convergence of the self-consistent field (SCF) cycle. The structure of compound was allowed to relax until the atomic force decreased by 0.01 eV/atom.Å. The optimized geometry was verified by vibrational analysis, which revealed that the structure exhibited real vibrational frequencies.

### 2.5. All-Atom Molecular Dynamic Simulation

Comprehensive all-atom Molecular Dynamic Simulation of AR, ERα and ERβ in complex with Ecdysterone was carried out using the PMEMD algorithm with the CUDA acceleration module integrated into Amber22 [18]. The topologies for target protein complexes were created using the antechamber [19] and tleap modules. The eight systems were immersed in a solvent environment generated with TIP3P [20], an explicit water model, inside a periodic box with atoms of the protein separated by at least 10 Å. The steepest descent approach with 2500 steps [21] was used to minimize energy in the systems. Position constraints were used to hold the protein in place, and the process was repeated six times, each time progressively reducing the degree of restraint. After the last restriction was lifted, the systems proceeded through one more round of energy minimization. To achieve the target temperature of 300 K, the systems were heated for 500 ps under a continuous volume, temperature (NVT) ensemble. The process was repeated twice, gradually reducing the positional limitations from 30 to 10 kcal mol. The system was equilibrated using a two-phase process: first under constant pressure (NPT) and then under constant volume (NVT). The NPT equilibration was performed in three steps over a total of 1.5 ns, with the temperature gradually increased to 300 K. This temperature increase was carried out in a controlled manner to ensure smooth thermal stabilization. Following this, the NVT equilibration was performed in seven steps over 3.5 ns, with the restraint weights gradually reduced in each step to allow the system to adapt to its natural dynamics. The final step of this phase was performed without any restraints, allowing the system to equilibrate freely at 300 K and 1 atm. Throughout the entire equilibration process, the system’s stability was closely monitored by tracking the convergence of key properties, such as temperature and pressure. The system was considered equilibrated when both the temperature and pressure exhibited stable, oscillating behavior within desired thresholds, indicating that optimal conditions for production runs had been achieved. Ultimately, six complexes total, a production run of 250 ns (2 µs) each, were executed under periodic boundary conditions. With the use of Langevin dynamics and isotropic position scaling, the temperature and pressure were controlled. The Particle Mesh Ewald (PME) approach was used to investigate long-range electrostatic interactions [22]. The SHAKE algorithm was employed to limit the interactions involving H atoms, and the non-bonded interactions were identified by using a cutoff of 10 Å [23]. The numerical integration was set up at two fs time steps. The simulated trajectories were examined using Chimera [24], VMD [25], and the CPPTRAJ [26] modules. As stability measurements, root mean square deviation (RMSD), root mean square fluctuation (RMSF), and radius of gyration (Rg) were considered.

### 2.6. Binding free energy

Free energy of binding ($\Delta G_{bind}$) was calculated by the Molecular Mechanics-Generalized Born (MM-GBSA) approach, where the last 50 ns trajectories (25 frames per ns) were used to perform calculations through the MMPBSA.py tools in the AMBER 22 package. Mathematically, the value of $\Delta G_{bind}$ was determined using Eq. 1, which takes into account the contributions of free energy in the gas term (Eq. 2), free energy in the solvation term (Eq. 3), and changes in entropy. The energy in gas term ($\Delta G_{gas}$) comprises electrostatic energy (${\Delta G}_{ele}$) and van der Waals energy (${\Delta G}_{vdW}$). The energy contribution to the solvation term ($\Delta G_{solv}$) is composed of the polar solvation energy (∆$G_{solv}^{ele}$) and the energy associated with the solvent-accessible surface area (∆$G_{solv}^{nonpolar}$) [27].


$$\Delta G_{bind}=\Delta G_{gas}+\Delta G_{solv}-T\Delta S$$ (1)

$$\Delta G_{gas}={\Delta G}_{vdW}+{\Delta G}_{ele}$$ (2)

$$\Delta G_{solv}={\Delta G}_{solv}^{ele}+{\Delta G}_{solv}^{nonpolar}$$ (3)

### 2.7. Principal Component Analysis (PCA)

Principal Component Analysis (PCA) was used to simplify the complex data from MD Simulation in order to reduce its dimensions and reveal important patterns and relationships within the dataset. Its objective also included examining conformational changes in proteins and deriving significant conclusions from the complex movements seen in MD trajectories. PCA was firmly grounded by aligning the MD trajectories as part of the default parameters. By diagonalizing the matrix, the Essential Dynamics (ED) approach was used to calculate the eigenvectors, eigenvalues, and their projections. The PCA module of the MDAnalysis [28] tool was used to do the analysis utilizing the two main components. In addition, the simulated system’s free energy profile was created using the data from these primary components.

### 2.8. Free Energy Landscape (FEL)

The free-energy landscape was constructed by using the gmx sham plugin of the GROMACS software package, after the conformational landscape and the molecular motions examined by the simulations were evaluated. The following formula illustrates potential protein conformations during molecular dynamics simulations in respect to Gibbs free energy based on the first two main components [29]


$$\Delta G=-K_{B}TlnP\left(PC_{1},{PC}_{2}\right)$$ (4)

The probability distribution of the molecular system, P(PC_1_, PC_2_), with its two principal components (PC_1_ and PC_2_), is given by an equation involving the Boltzmann constant (K_B)_ and absolute temperature (T). P(PC_1_, PC_2_) represents the system’s conformational states along these principal components, obtained through principal component analysis (PCA). PCA identifies the directions of maximum variance in the molecular conformations. Collectively, these components describe the free energy landscape, visualizing the system’s energy distribution. This landscape provides insights into the kinetics and thermodynamic stability of the molecules by revealing different energy states and their corresponding conformational probabilities. The free energy change, ∆G, further elucidates the system’s stability based on these conformational states.

## 3. Results and discussion

### 3.1. Molecular Binding analysis of androgen receptor

**Fig 2:**
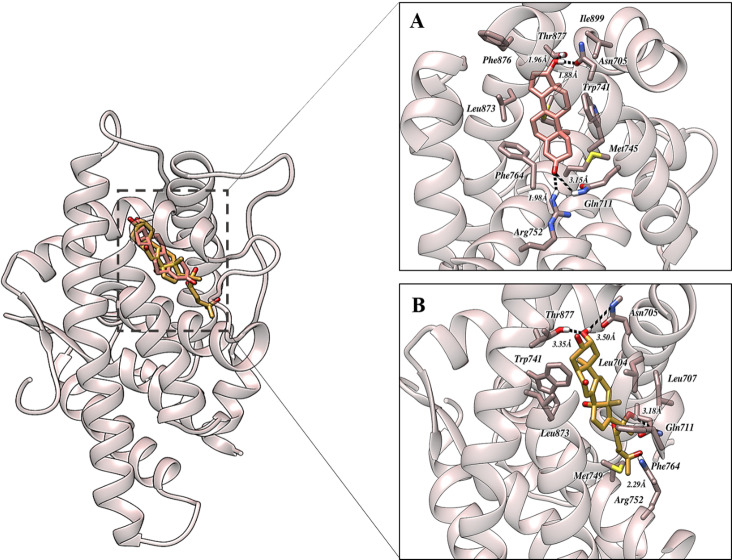
The binding orientation of AR in complex with A) Testosterone (native ligand) and B) Ecdysterone. The dashed black line represents hydrogen bonding. Pictures were rendered utilizing UCSF Chimera.

The AR – testosterone complex (Fig 2A) exhibited a network of multiple hydrogen bonds and hydrophobic interactions. The oxygen atom of the carbonyl group in testosterone formed two hydrogen bonds with the amine groups of Gln711 and Arg752 with bond lengths of 1.98 and 3.15Å respectively. The hydrogen bond with Gln711 bond suggest a more optimal alignment of the dipoles between the carbonyl oxygen and the amine nitrogen, enhancing the stability of this interaction. Additionally, the oxygen atom of Asn705 formed a hydrogen bond with the hydroxyl group of testosterone, spanning a distance of 1.88 Å. Another hydrogen bond was observed between the oxygen atom of testosterone and the hydroxyl group of Thr877, spanning at a distance of 1.96 Å. These hydrogen bonds exhibit strong dipole-dipole interactions, driven by significant electronegativity differences between oxygen, nitrogen, and hydrogen, generating partial charges and dipole moments. Moreover, hydrophobic interactions were also noted, involving Trp741, Met745, Phe764 and Leu873. Furthermore, π–π stacking was identified between the core of testosterone and Trp741, leads to contribution of the stability of testosterone within the binding region of AR. The AR in complex with testosterone displayed with comparable types of interactions as AR_Ecdysterone exhibited. This suggests that both molecules may stabilize the receptor similarly, potentially leading to similar effects on its function. The binding affinity was found to be -8.65 kcal/mol.

To determine the optimal orientation of Ecdysterone within the active site of AR, docking simulation was performed. The simulation revealed strong binding interactions between Ecdysterone and AR, with a docking score of -8.97 kcal/mol. Molecular docking results demonstrated (Fig 2B) that Ecdysterone formed a stable complex within the binding region of AR. The key residues in the active configuration of AR, namely Asn705, Gln711, Trp741, Arg752, Phe764, Leu873 and Thr877 play a crucial role in AR activation. Inter-molecular interaction analysis during molecular simulation exhibited revealed multiple intermolecular hydrogen bonds, representing strong dipole-dipole interactions. The amine group of Gln711 formed a hydrogen bond with hydroxyl group of Ecdysterone spanning at a distance of 3.18 Å. The amine group of Arg752 established a bond with another hydroxyl group at a distance of 2.29 Å. The hydroxyl group of Thr877 formed a bond with oxygen atom of Ecdysterone, spanning at a distance of Å. Additionally, the NH2 group of Asn705 established a hydrogen bond spanning at a distance of 3.50 Å. In the active configurations of the AR, Asn705, Gln711 and Arg752 consistently form hydrogen bonds. In addition, additional stability was achieved through a face-to-face interaction between phenanthrene core of Ecdysterone and Trp741. This interaction, along with other hydrophobic interactions, played a crucial role in stabilizing Ecdysterone within the binding region of AR. The hydrophobic interactions consistently involved key residues such as Pro682, Leu704, Gln711, Trp741, Met745, and Phe764. These residues created a hydrophobic pocket that helped anchor Ecdysterone firmly within the binding site, enhancing the overall stability and specificity of the interaction. These interactions are presented in a tabular format is provided in supplementary information as S1 Table.

### 3.2. Molecular Binding Analysis of Estrogen Receptor (Alpha)

**Fig 3:**
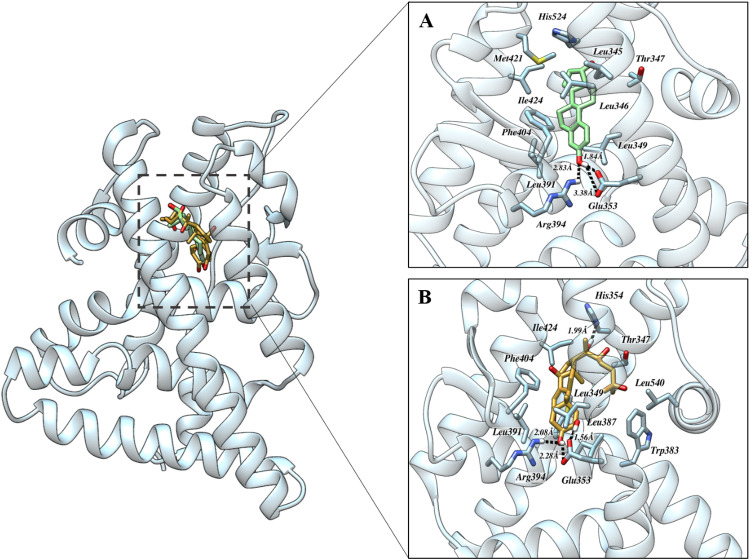
The binding orientation of ER α in complex with A) Estradiol (native ligand) and B) Ecdysterone. The dashed black line represents hydrogen bonding. Pictures were rendered utilizing UCSF Chimera.

The ERα in complex with Estradiol (Fig 3A) established multiple sources of interaction, including hydrogen bonding and hydrophobic interaction and likely dipole-dipole interactions between polar groups. The amine group of Arg394 established a hydrogen bond with the hydroxyl group of Estradiol spanning at a distance of 2.83 Å. On the other hand, two oxygen atoms of Glu353 firmly involved in making a bond with the hydroxyl group of Estradiol spanning at a distance of 1.84, 3.38 Å. In this context, His524 forms hydrophobic interactions with the core of estradiol within the binding cleft, while Phe404 induces π-π interactions with estradiol. Resulting hydrophobic interaction was introduced by Leu345, Leu346 and Ile424 contributing to the stability and specificity of the ERα-estradiol complex, which is vital for mediating the biological effects of estrogens. The binding affinity was determined to be -8.92 kcal/mol.

The complex of ERα with Ecdysterone (Fig 3B) demonstrated a sophisticated network of molecular interactions, including hydrogen bonding, hydrophobic interactions, and electrostatic forces and dipole-dipole interactions contributing to the binding stability and specificity. The amine group of Arg394 formed a stable hydrogen bond with hydroxyl group of Ecdysterone spanning at a distance of 2.08 Å. The hydrogen bond between the amine group of Arg394 and the hydroxyl group of Ecdysterone is a strong dipole-dipole interaction driven by the significant electronegativity differences between oxygen, and hydrogen. Additionally, the hydroxyl group of Ecdysterone engaged in hydrogen bonding with two distinct oxygen atoms of Glu353, forming bonds at distances of 1.56 Å and 2.28 Å, respectively. Another hydroxy group established a bond with Glu305 occurring bond length 1.56 Å. The nitrogen atom of His524 formed a bond with hydroxyl group of Ecdysterone at a distance of 1.99 Å. In addition to these hydrogen bonds, a series of hydrophobic interactions were identified, which are crucial for stabilizing the hydrophobic core of the binding pocket. Notable residues involved in these interactions include Leu349, Leu387, Leu391, Ile424, and Phe404. These residues create a non-polar environment that favors the binding of Ecdysterone, further enhancing the stability of the ERα-Ecdysterone complex. The bonding energy was found to be -9.68 kcal/mol.

### 3.3. Molecular Binding Analysis of Estrogen Receptor (Beta)

**Fig 4:**
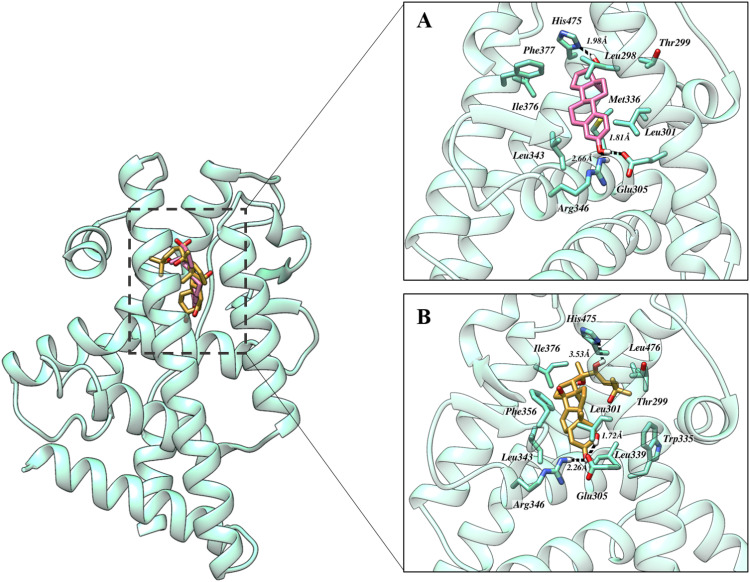
The binding orientation of ERβ in complex with A) Estradiol (native ligand) and B) Ecdysterone. The dashed black line represents hydrogen bonding. Pictures were rendered utilizing UCSF Chimera.

The ERβ - estradiol (Fig 4A) complex exhibited a network of hydrogen bonds, including a hydrogen bond between the hydroxyl group of estradiol and the amine group of Arg346 at a distance of 2.66 Å. Additionally, the oxygen atom of Glu305 formed a bond with the hydroxyl group of estradiol at a bond length of 1.81 Å. Furthermore, the nitrogen atom of the pyridine ring from His475 established a bond with another hydroxyl group of estradiol at a distance of 1.98 Å. This short bond length indicates a strong dipole-dipole interaction due to the significant electronegativity difference between nitrogen and oxygen. Notably, the interactions involving Leu298, Thr299, Leu301, Met336, Ile376, and Phe377 contribute significantly to the stability and specificity of the ERβ-estradiol complex. Notably, the ERβ-Ecdysterone complex exhibited a similar pattern of interactions as observed in the ERβ-estradiol complex. The binding affinity was found to be -9.26 kcal/mol.

The ERβ complexed with Ecdysterone (Fig 4B) exhibited a well-defined network of hydrogen bonds within the binding region, analogous to those observed in ERβ. The oxygen atom of Glu305 formed a hydrogen bond with the hydroxyl group of Ecdysterone at a distance of 1.72 Å. This interaction, characteristic of a strong dipole-dipole interaction, arises from the substantial difference in electronegativity between oxygen and hydrogen. In addition, the same hydroxyl group established another hydrogen bond with the amine group of Arg346, with a bond length of 2.26 Å. The nitrogen atom of the pyridine ring in His475 was also involved in hydrogen bonding with Ecdysterone, although at a longer distance of 3.53 Å, which may suggest a weaker but still significant interaction. This bond could contribute to the overall orientation and stabilization of Ecdysterone within the binding pocket. Beyond hydrogen bonds, several other residues were implicated in electrostatic and hydrophobic interactions, which further stabilized the complex. Additionally, Phe356 forms a π-π interaction with the scaffold of Ecdysterone. The keynote residues involved in these interactions include Leu343, Thr299, Leu301, Leu339, Met340, Phe356, and Ile376. These residues establish a hydrophobic environment that facilitates the binding of Ecdysterone within the ligand-binding domain of ERβ. The binding affinity was found to be -10.89 kcal/mol.

### 3.4. DFT-based analysis

**Fig 5:**
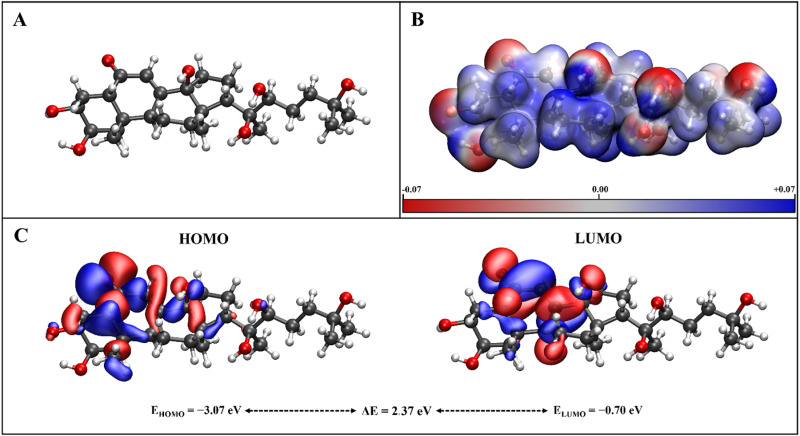
A visualization of the electronic properties of Ecdysterone. (A) The optimized structure, (B) molecular electrostatic potential map (isovalue = 0.01e), and (C) HOMO-LUMO representation (isovalue = 0.015e).

To study the electronic properties of Ecdysterone at molecular level, its optimized structure, as shown in **Fig 5A** was utilized for the DFT-based analyses. The molecular electrostatic potential (MESP) map is a valuable representation to study the electrophilic and nucleophilic properties of a compound by considering its electronic density over various sites in the structure. In the MESP map of Ecdysterone, depicted in **Fig 5B**, the red and blue contours represent electron-rich and electron-deficient sites, respectively, while the regions with white contours show the neutral sites. It can be seen from the MESP isosurface representation of Ecdysterone that nucleophilic sites are localized at oxygen atoms while the electrophilic sites are around hydrogen atoms, as evident from the electronegativities of these elements as compared with that of carbon. Furthermore, the spatial distribution of frontier molecular orbitals i.e., the highest occupied (HOMO) and the lowest unoccupied (LUMO) molecular orbitals, was used to estimate the chemical reactivity and intra-molecular charge transfer sites in the structure of Ecdysterone. The HOMO-LUMO distribution in Ecdysterone is illustrated in **Fig 5C**, where the red and blue isosurfaces represent negatively and positively charged sites. An appropriately large HOMO-LUMO energy gap (E) of 2.37 eV indicates the kinetic stability of this molecule, while the presence of HOMO and LUMO isosurfaces at the same site of the molecule shows low intramolecular charge transfer.

### 3.5. Assessment of structural stability

The Root Mean Square Deviation (RMSD) is a quantitative measure that is used to evaluate the deviation between a protein’s native and final conformations during molecular dynamics simulations, providing valuable insights into the protein’s stability and dynamic behavior. In structural biology, RMSD is an essential parameter for evaluating the stability of simulated bio-molecular systems, where generally, lower values signify greater stability and higher values indicate less stable complexes.

**Fig 6:**
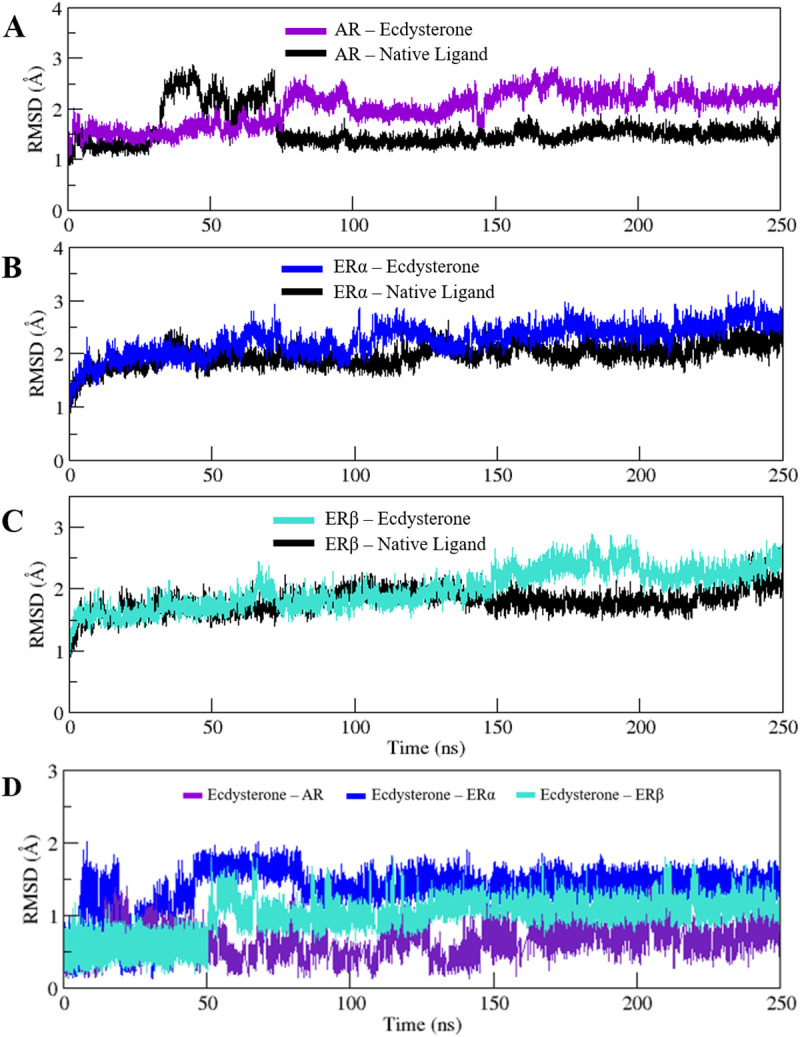
RMSD plots of the 250 ns simulated trajectories of A) AR B) ERα and C) ERβ in complex with Ecdysterone and their respective native ligands. (D) RMSD plots of the 250 ns simulated trajectories of Ecdysterone in complex with AR, ER α ERβ.

The RMSDs of the 250 ns simulated trajectories of each complex were computed in order to assess the stability of the structure and fundamental dynamics of AR, ERα and ERβ in complex with Ecdysterone when compared with the native complex. As can be observed in **Fig 6A**, AR in complex with Ecdysterone showed deviations in terms of RMSD when compared with the native complex, while ERα (**Fig 6B**) and ERβ (**Fig 6C**) in complex with Ecdysterone showed consistent RMSDs with minimal deviations when compared with their respective native complexes. AR in complex with Ecdysterone demonstrated an average RMSD of 2.0 ± 0.34 Å compared with 1.56 ± 0.34 Å observed when bound to its native ligand. ERα and ERβ in complex with Ecdysterone exhibited an average RMSD of 2.26 ± 0.28 and 1.98 ± 0.31 Å when compared with 1.96 ± 0.20 and 1.97 ± 0.17 Å as observed when bound to their respective native complexes. All the systems demonstrated an approximate projected deviation value of around 2 Å, ERα and ERβ however, demonstrated the most consistent RMSDs with respect to their native complex structure, suggesting stability of the protein-ligand complex. RMSDs of the 250ns simulated trajectories of the three complexes are also presented overlapped with each other in Supplementary S2(A) Fig for the ease of comparison. Further analysis of the trajectories was also performed to assess the stability of Ecdysterone bound to AR, ERα and ERβ. As depicted in **Fig 6D** Ecdysterone as part of complex with ERβ displays the most stability with minimal deviations after the 50 ns mark.

### 3.6. Analysis of structural flexibility

To assess the intrinsic flexibility of the AR, ERα, and ERβ residues over a 250 ns MD simulation, Root Mean Square Fluctuations (RMSF) were calculated from the simulated trajectories. In a biomolecular system, higher RMSF values indicate greater flexibility and a less stable state, while lower fluctuation levels suggest increased stability.

**Fig 7:**
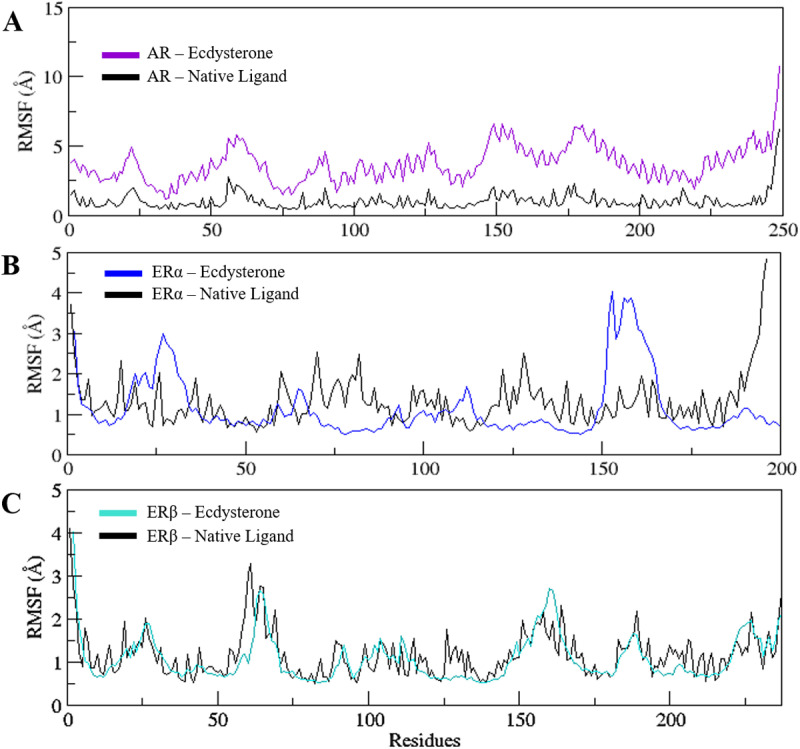
RMSF plots of the 250 ns simulated trajectories of A) AR and B) ERα C) ER β with Ecdysterone and native ligands.

As shown in the RMSF plots for AR (Fig 7A) high fluctuations were observed, indicating instability and also in case of ERα (Fig 7B) displaying a disruption of the target protein binding residues when complexed with Ecdysterone. The average RMSF values for AR and ERα were recorded as 3.70 ± 1.32 Å and 1.10 ± 0.68 Å, respectively, compared to their native ligands, displaying average values of 1.02 ± 0.62 Å and 1.29 ± 0.58 Å. The RMSF plot for ERβ (Fig 7C), however showed consistent fluctuations with minimal variations, suggesting a stable protein-ligand complex. The average RMSF values for ERβ and its native ligand were 1.07 ± 0.52 Å and 1.19 ± 0.61 Å, respectively. RMSF plots of the 250ns simulated trajectories of the three complexes along with the native complex are also presented overlapped with each other in Supplementary S2(B) Fig for the ease of comparison.

### 3.7. Evaluation of structural compactness

Further study was carried out on the time-dependent convergence of the radius of gyration (Rg), which describes the structural characteristics of simulated ensembles. The root-mean-squared distance between the protein’s constituent parts and its center of mass is known as Rg, and it indicates how compact the protein is. After evaluating the molecular effects of ligand binding on the receptor, we used Rg to investigate the folding dynamics of the protein across time. Rg values fluctuate over time in an improperly folded conformation, but stable gyration values are usually maintained by a properly folded conformation.

**Fig 8:**
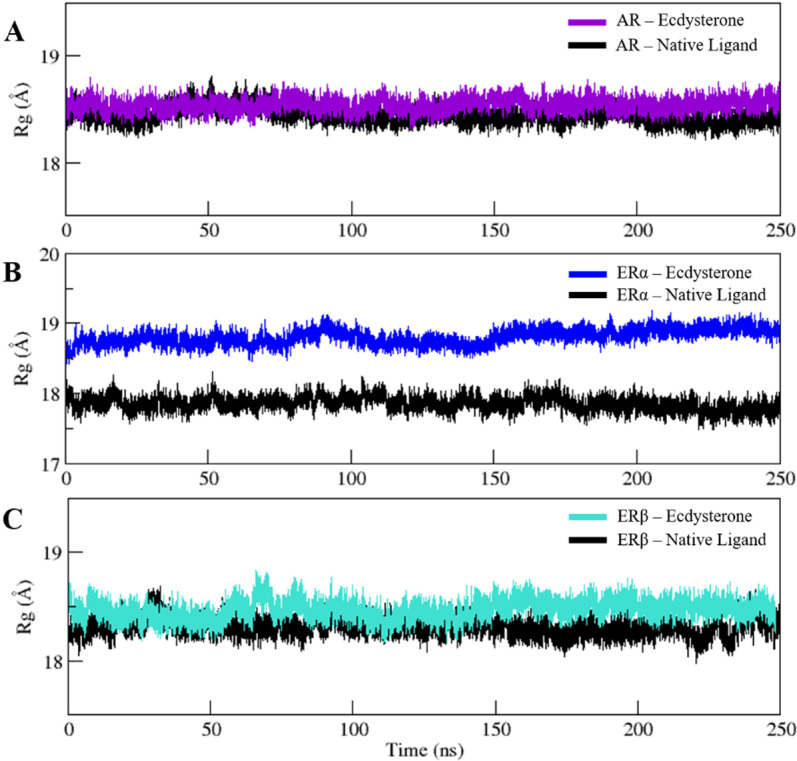
Rg plots of the 250 ns simulated trajectories of A) AR and B) ERα and C) ERβ in complex with Ecdysterone and their respective native ligands.

As can be observed in **Fig 8B** and **8C**, both AR and ERβ displayed minimal fluctuations in terms of Rg in complex with Ecdysterone, displayed average values of 18.54 ± 0.05, 18.44 ± 0.08 Å, compared with the native ligands which displayed an average value of 18.44 ± 0.07 and 18.38 ± 0.08 Å, respectively, suggesting structural compactness rendering stability to the protein complex. ERα in complex with Ecdysterone, however, displayed an average Rg vale of 18.80 ± 0.10 Å, demonstrating high fluctuations when compared with 17.85 ± 0.09 Å of its native as shown in **Fig 8A**. Rg plots of the 250ns simulated trajectories of the three complexes along with the native complex are also presented overlapped with each other in Supplementary S3(C) Fig for the ease of comparison.

### 3.8. Dynamics of receptor-ligand hydrogen bonding

**Fig 9:**
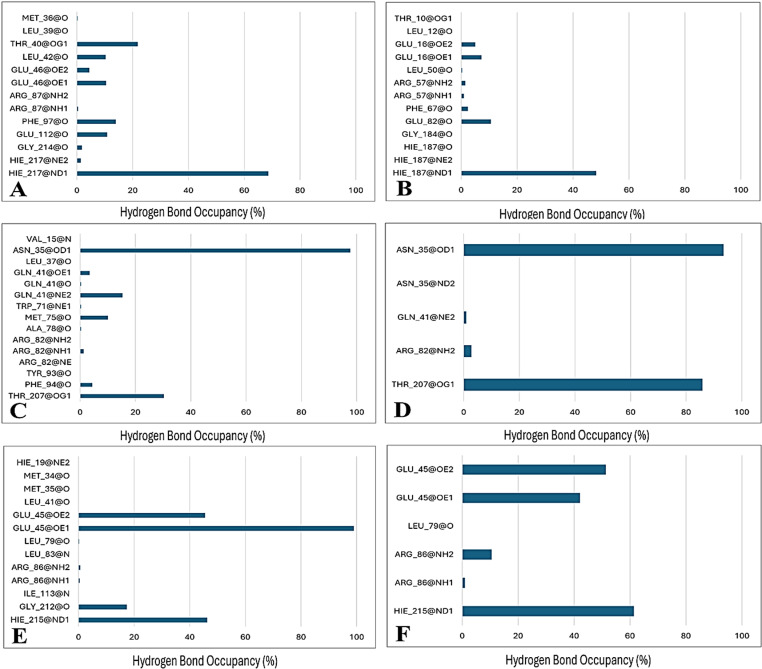
Dynamic occupancy of hydrogen bonding between ligands and various residues of protein in A) ERα-Ecdysterone, B) ERα-Native Ligand, C) AR-Ecdysterone, D) AR-Native Ligand, E) ERβ-Ecdysterone and F) ERβ -**Native Ligand systems.**

The occupancy of hydrogen bonding was monitored throughout the MD trajectories using the CPPTRAJ software for exploring the most interacting receptor residues with the ligands through hydrogen bonding [30] Dynamic patterns of receptor-ligand hydrogen bonding for all of the studied systems are presented in **Fig 9**. In the ERα system, a greater occupancy of hydrogen bonds can be seen by Ecdysterone as compared to the native ligand with the receptor residues. The occupancy of hydrogen bonds and number of significantly interacting amino acid residues with Ecdysterone also predominates in the AR systems. Similarly, Ecdysterone also predominates in the case of ERβ. In can be concluded from these observations that Ecdysterone is better able to interact with the active site residues by forming hydrogen bonds of greater occupancy as compared to the native ligands. Furthermore, if all the Ecdysterone-bound systems are compared with each other, a better consistency of hydrogen bonds is seen in the ERβ system both in terms of number and occupancy of the hydrogen bonds formed with the pocket residues.

### 3.9. Evaluation of binding affinity

The determination of binding free energies ($\Delta G_{bind}$) is an essential step in structure-based drug design for understanding the interaction between a ligand and a protein. In this study, the $\Delta G_{bind}$ for all the protein-ligand complexes was calculated using 1000 snapshots from MD trajectories, applying the MM-GBSA equation to provide a more comprehensive understanding of the interaction network that drive complex formation A higher negative value of $\Delta G_{bind}$ indicates a stronger affinity between the ligand and receptor, whereas a less negative value suggests a lower affinity and therefore less persistent binding. **Table 1** presents the computed free energy of Ecdysterone with AR, ERα, ERβ and compared with their respective native ligands. The results show that the primary factor favoring the binding process is the ability of the compounds to form van der Waals and electrostatic interactions with the target proteins. AR – Ecdysterone complex showed the highest $\Delta G_{bind}$ followed by ERβ – Ecdysterone complex featuring the values of –53.76 and –41.85 kcal/mol respectively, while ERα – Ecdysterone complex displayed a value of –47.12 kcal/mol. A detailed analysis of the individual energy components contributing to the binding process was also conducted. The decomposition of total energy revealed that the non-electrostatic interactions, such as van der Waals (Evdw) and electrostatic interactions (Eele), were primarily favorable. However, the energy contribution to the solvation were found to be unfavorable for binding across the tested protein-ligand complexes. This comprehensive binding energy analysis suggests stable and favorable energetic mechanisms driving the molecular recognition between Ecdysterone and the target receptors. However, interesting to note here is that while the $\Delta G_{bind}$ displayed for ERα – Ecdysterone was found most favorable out of all studied complexes, the overall effects of this energy might have caused overall negative effects on the binding with the target protein, as evident by the high fluctuations observed in **Fig 7B**.

**Table 1:** Contributions of various energetic terms to the binding free energy ($\Delta G_{bind}$) of the studied protein-ligand complexes, provided as mean ± standard error of the mean. All units are in kcal/mol.

	Systems	${\Delta G}_{vdW}$	${\Delta G}_{ele}$	${\Delta G}_{solv}^{ele}$	${\Delta G}_{solv}^{nonpolar}$	$\Delta G_{bind}$
1	**AR – Ecdysterone**	−68.45 ± 0.07	−22.49 ± 0.14	45.94 ± 0.12	−8.76 ± 0.01	−53.76 ± 0.10
2	**AR – Native Ligand**	−45.37 ± 0.06	−19.75 ± 0.06	24.83 ± 0.04	−5.66 ± 0.01	−45.95 ± 0.06
3	**ERα** – **Ecdysterone**	−62.80 ± 0.09	−21.38 ± 0.21	45.25 ± 0.18	−8.19 ± 0.01	−47.12 ± 0.10
4	**ERα – Native ligand**	−41.49 ± 0.06	−6.53 ± 0.09	16.36 ± 0.06	−5.17 ± 0.01	−36.83 ± 0.07
5	**ERβ – Ecdysterone**	−60.82 ± 0.09	−31.95 ± 0.16	50.00 ± 0.12	−8.28 ± 0.01	−51.06 ± 0.09
6	**ERβ – Native Ligand**	−41.88 ± 0.07	−20.28 ± 0.09	25.76 ± 0.05	−5.45 ± 0.01	−41.85 ± 0.07

### 3.10. Conformational Motions and Thermodynamic Landscape

**Fig 10:**
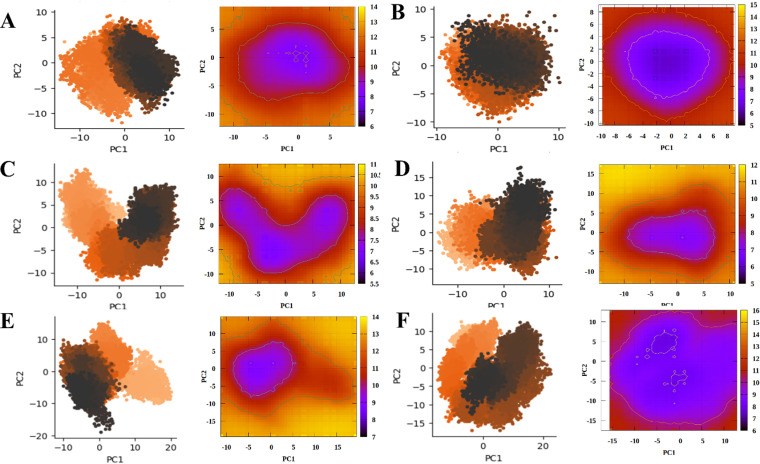
A side-by-side representation of the PCA and Free Energy Landscape (FEL) profiles of A) AR - Ecdysterone B) AR - Native Ligand C) ERα - Ecdysterone D) ERα - Native Ligand E) ERβ - Ecdysterone F) ERβ - Native Ligand.

To further investigate the stability and conformational changes in ligand-bound protein systems, principal component analysis (PCA) and free energy landscape (FEL) profiles were generated from MD trajectories. A side-by-side comparison of the PCA and FEL profiles for all systems is presented in **Fig 10**. In the AR systems (**Figs 10A-10B**), both complexes exhibit conformational stability, evidenced by a single large energy basin near the minimal variability regions of PC1 and PC2. In contrast, the ERα system complexed with ecdysterone (Fig 10C) shows a loss of stability, as indicated by the formation of multiple stable energy states, unlike its complex with the native ligand (Fig 10D), which remains stable. The ERβ-Ecdysterone complex (Fig 10E) displayed increased stability, as shown by a smaller energy basin centered around 0 (PC1) and -5 (PC2). In contrast, the native ligand complex (**Fig 10F**) exhibits a broader range of conformations, suggesting the presence of multiple metastable states. This indicates that Ecdysterone confers stability to the ERβ complex. These findings suggest that Ecdysterone stabilizes both AR and ERβ systems, while it decreases the stability of ERα. The results are consistent with the conclusion that Ecdysterone acts as a strong binder to AR and ERβ receptors.

## 4. Conclusion

This study employed a comprehensive computational framework to explore the chemical properties and inter-molecular interactions of Ecdysterone, focusing on its binding with the Androgen Receptor (AR), Estrogen Receptor alpha (ERα), and Estrogen Receptor beta (ERβ). Through all-atom molecular dynamics simulations spanning 250 ns for each system, the results aligned with previously reported experimental findings, strongly supporting the hypothesis that Ecdysterone preferentially binds to ERβ, competing with its native ligand over ERα and AR. Our results demonstrate that the binding of Ecdysterone with ERα, exhibited continuous favorability in all the stability measures that were used for this study. The stability and compactness of the Ecdysterone – ERβ complex, characterized by minimal per-residue fluctuations, and high hydrogen bonding occupancy further underscore this preference. Interestingly, while binding free energy calculations initially indicated more favorable interactions with ER-α, this was contradicted by high fluctuations, likely reducing the overall binding efficacy. Additionally, machine learning-based principal component analysis revealed consistent motion, and free energy profiles confirmed stable energy basins with minimal variations for the ERβ complex. In conclusion, this study, utilizing in-silico methods, confirms the experimental claims suggesting that Ecdysterone’s anabolic effects are mediated primarily through ERβ, distinguishing it from traditional anabolic agents that interact with AR. These findings not only validate existing claims but also establish a foundation for future research into Ecdysterone’s potential therapeutic applications and associated risks. Future research on ecdysterone should be focused on its long-term safety, potential side effects, and therapeutic applications beyond its anabolic properties. Investigating its effects on hormone regulation, metabolic pathways, and potential off-target interactions will be crucial for assessing its suitability as a performance enhancer or therapeutic agent. Additionally, large-scale clinical studies are also needed to validate in-silico and preclinical findings, ensuring a comprehensive understanding of its efficacy and risks.

## Supporting information

Fig S1 The chemical structures of Ecdysteroid and the native ligands of AR, ERα, and ERβ. (TIF)

Fig S2 A) RMSD plots of the 250 ns simulated trajectories of AR, ERα and ERβ in complex with Ecdysterone. **B)** RMSF plots of the 250 ns simulated trajectories of AR, ERα and ERβ in complex with Ecdysterone. **C)** Rg plots of the 250 ns simulated trajectories of AR, ERα and ERβ in complex with Ecdysterone.
(TIF)

Table S1 Molecular Binding Interactions of Ecdysterone with AR, ERα, and ERβ. (DOCX)
