# Supplementary figures and images for: Molecular insights into the role of Estrogen Receptor Beta in Ecdysterone Mediated Anabolic Activity
Source: PLoS One. 2025 Jun 2;20(6):e0320865. doi: 10.1371/journal.pone.0320865 (PMC12129227; doi:10.1371/journal.pone.0320865)

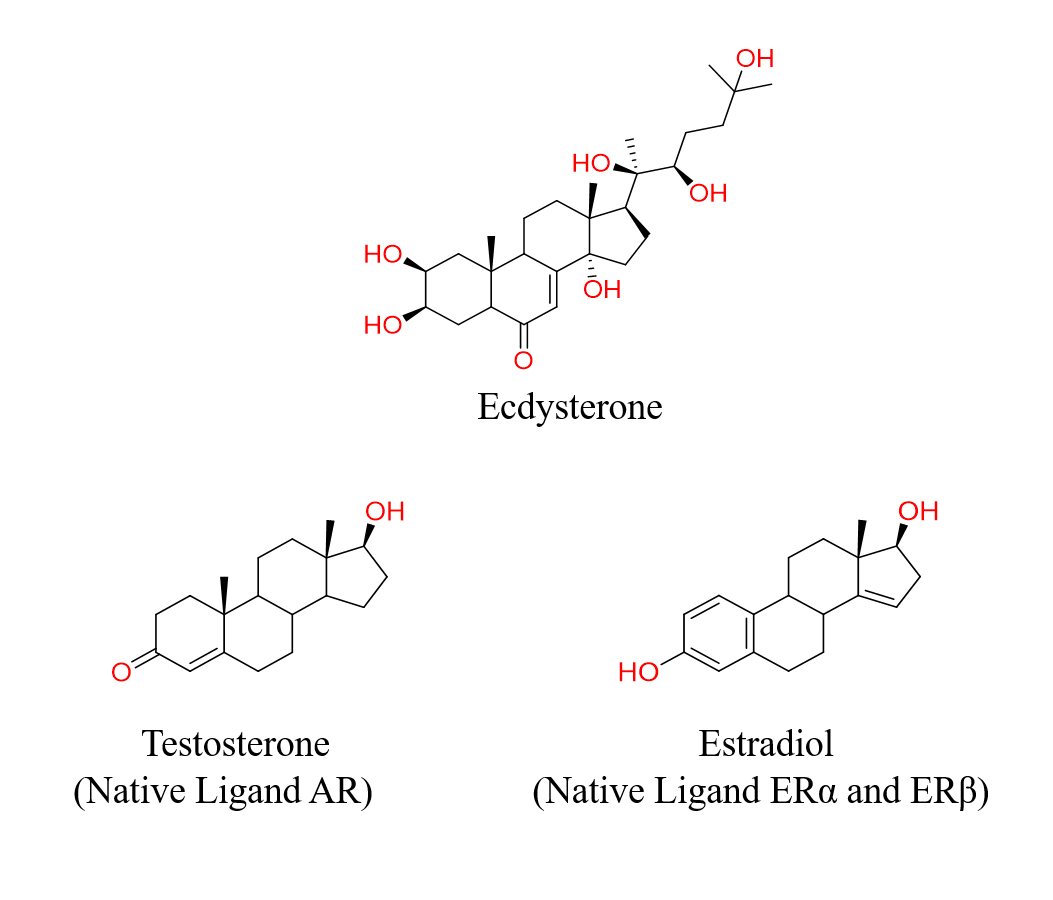

Supplement: Fig S1 — (TIF) [file pone.0320865.s001.tif]

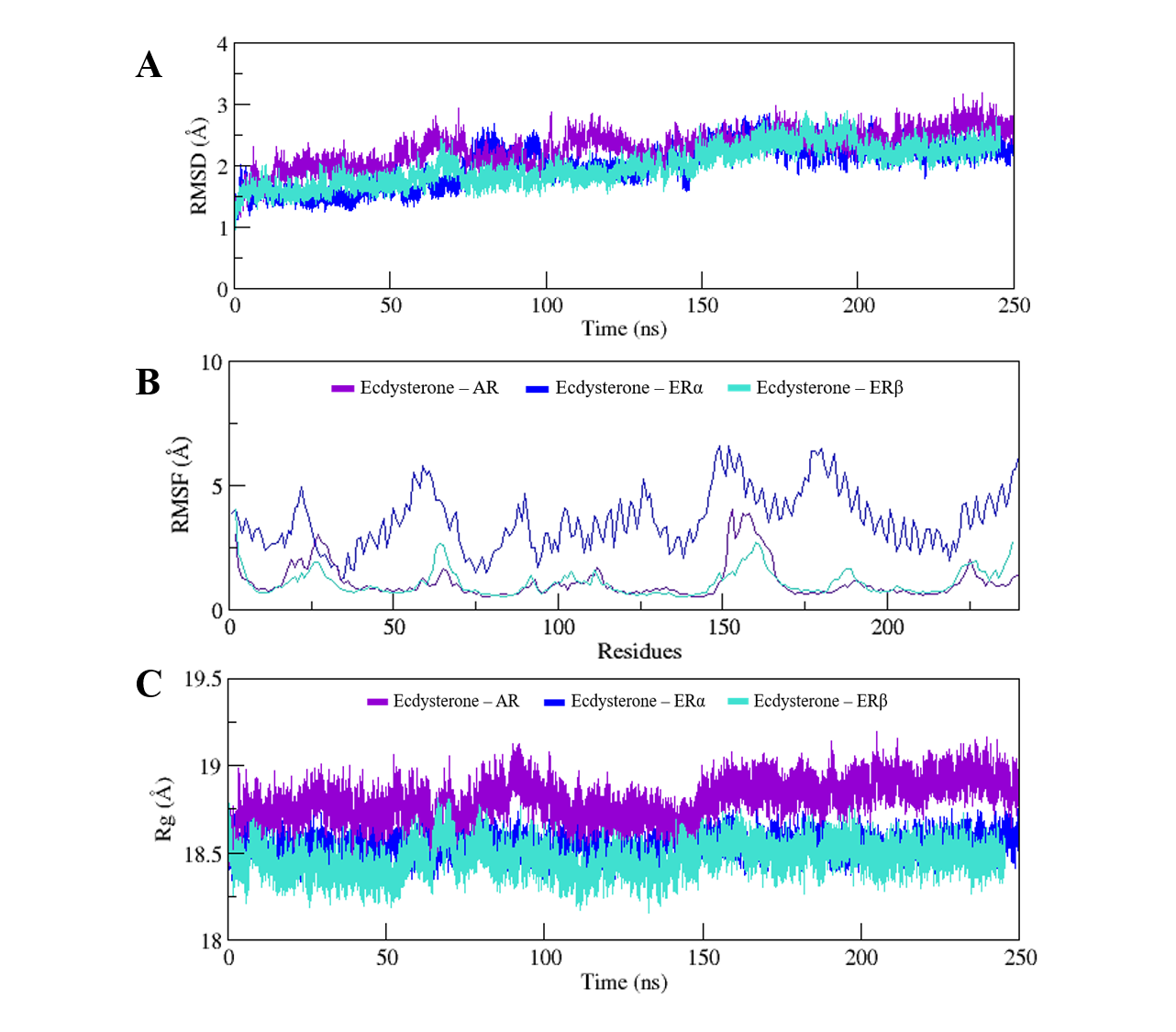

Supplement: Fig S2 — B) RMSF plots of the 250 ns simulated trajectories of AR, ERα and ERβ in complex with Ecdysterone. C) Rg plots of the 250 ns simulated trajectories of AR, ERα and ERβ in complex with Ecdysterone. (TIF) [file pone.0320865.s002.tif]
