# Supplementary material for: Molecular insights into the role of Estrogen Receptor Beta in Ecdysterone Mediated Anabolic Activity
Source: PLoS One. 2025 Jun 2;20(6):e0320865. doi: 10.1371/journal.pone.0320865 (PMC12129227; doi:10.1371/journal.pone.0320865)
Supplement: Table S1 — (DOCX) [file pone.0320865.s003.docx]

**Table S1:** Molecular Binding Interactions of Ecdysterone with AR, ERα, and ERβ.

| **Compounds** | **AR** | **Score**  **Kcal/mol** | **ERα** | **Score Kcal/mol** | **ERβ** | **Score Kcal/mol** |
| --- | --- | --- | --- | --- | --- | --- |
|  | **Interaction Pattern Analysis** |  | **Interaction Pattern Analysis** |  | **Interaction Pattern Analysis** |  |
| Ecdysterone | HB: Asn705, Gln711, Arg752, and Thr877  Hydro: Pro682, Leu704, Gln711, Trp741, Met745, and Phe764 | -8.97 | HB: Arg394, Glu353, Glu305, and His524.  Hydro: Leu349, Leu387, Leu391, Ile424, and Phe404. | -9.68 | HB: Glu305, Arg346, and His475  Hydro: Leu343, Thr299, Leu301, Leu339, Met340, Phe356, and Ile376 | -10.89 |
| AR_Testosterone (Native) | HB: Asn705, Gln711, Arg752, and Thr877  Hydro: Trp741, Met745, Phe764 and Leu873 | -8.65 | - | - | - | - |
| ERα _Estradiol (Native) | - | - | Arg394, Glu353 and His524  Hydro: Leu346, Leu349, Leu387, Leu391, Ile424, and Phe404 | -8.92 | - | - |
| ERβ _Estradiol (Native) | - |  | - | - | HB: Glu305, Arg346 and His475  Hydro: Leu298, Thr299, Leu301, Met336, Ile376, and Phe377 | -9.26 |
|  |  |  |  |  |  |  |
